# *Fusarium* infection complicating rheumatic keratitis that acutely progressed to endophthalmitis during regular infusion of tocilizumab: a case report

**DOI:** 10.1186/s12886-021-01981-9

**Published:** 2021-05-19

**Authors:** Yusuke Mitsuoka, Takeshi Soma, Kazuichi Maruyama, Kohji Nishida

**Affiliations:** 1 Department of Ophthalmology, Osaka University Graduate School of Medicine, Suita, Osaka, Japan; 2 Department of Vision Informatics, Osaka University Graduate School of Medicine, Suita, Osaka, Japan

**Keywords:** *Fusarium* keratitis, Endophthalmitis, Tocilizumab, Electroretinogram, Case report

## Abstract

**Background:**

Filamentous fungi are ubiquitous in plants, water, and soil. The predominant fungi that infect the human cornea include *Fusarium* and *Aspergillus* species. The onset of fungal endophthalmitis is indolent, and typically takes weeks to months to develop after corneal infection. We report a case of *Fusarium* infection complicating rheumatic keratitis that acutely progressed to endophthalmitis during intravenous tocilizumab therapy.

**Case presentation:**

A 65-year-old female patient was referred to our department due to pain and decreased vision in her left eye. Slit-lamp examination showed a white focus on the upper peripheral cornea, hypopyon, anterior chamber fibrin formation, marked ciliary hyperemia, and whole corneal epithelial defects. As the corneal scraping smear was positive for filamentous fungi and *Fusarium* species were detected by aqueous humor polymerase chain reaction, anti-fungal therapy was started. Although the initial response to anti-fungal therapy was good, we observed corneal infiltration, worsening hypopyon, and vitreous opacity after tocilizumab infusion. Given that the infection continued to progress despite conservative therapy, we performed penetrating keratoplasty combined with vitrectomy. After removal of the white focus beneath the intraocular lens, a temporary corneal prosthesis was mounted and the dense vitreous opacity was removed. Finally, a frozen donor graft was sutured in place. The corneal infiltration, hypopyon, and vitreous opacity all disappeared after the operation.

**Conclusion:**

The rapid progression of *Fusarium* keratitis to endophthalmitis in a patient who was receiving a regular infusion of tocilizumab demonstrates that ocular condition should be closely monitored during systemic tocilizumab administration due to increased risk of infection.

## Background

Filamentous fungi are ubiquitous; they are present in plants, water, and soil. *Fusarium* species and *Aspergillus* species are the main fungi that infect the human cornea. The major route of infection is corneal trauma, and the most frequent cause of traumatic fungal endophthalmitis is filamentous fungal infection [1]. Contact lens wear, corneal transplantation, and steroid use have also been reported as causes of infection [2].

The clinical features of a typical filamentous fungal keratitis include a grayish-white ulcer accompanied by peripheral feathery corneal infiltrate, endothelial plaque formation, and hypopyon. Resistance to antifungal agents is frequently encountered and surgical treatment, including corneal transplantation, is sometimes required [3].

Severe fungal keratitis can lead to endophthalmitis [4, 5]. The onset of fungal endophthalmitis is typically indolent, and occurs weeks or months after corneal infection. We report a case of *Fusarium* infection complicating rheumatic keratitis that acutely progressed to endophthalmitis while the patient was receiving regular infusion of the anti-interleukin-6 (IL-6) receptor antibody tocilizumab to treat rheumatoid arthritis.

## Case presentation

A 65-year-old female patient was referred to our department due to pain and decreased vision in her left eye. Her medical history included rheumatoid arthritis, for which she received monthly intravenous tocilizumab injections (8 mg/kg). There was no family history. She was followed by her previous doctor for secondary glaucoma and recurrent rheumatic keratitis. She had a prior history of bilateral cataract surgery.

The patient was aware of the pain in her left eye 10 days before admission. At this time, 0.1% betamethasone 4 times daily and 0.1% tacrolimus 2 times daily were continued for the treatment of rheumatic keratitis. Five days before the patient came to our department, slit-lamp examination at the previous clinic showed a white stromal infiltrate on the peripheral cornea at the 12 o’ clock position. Since exacerbation of rheumatic keratitis was suspected, oral prednisolone (10 mg) was started, dexamethasone was administered via subconjunctival injection, and 0.1% betamethasone administration was increased to 6 times daily.

Three days before admission to our department, when the patient first experienced decreased vision, the stromal infiltrate in her left eye worsened and a hypopyon emerged. At this point, microbial keratitis was suspected, and hourly topical 0.5% moxifloxacin, hourly 0.3% tobramycin, and systemic ceftriaxone (2 g every 24 h) were prescribed, whereas oral prednisolone was withdrawn and 0.1% betamethasone administration was reduced to 3 times daily.

**Fig. 1:**
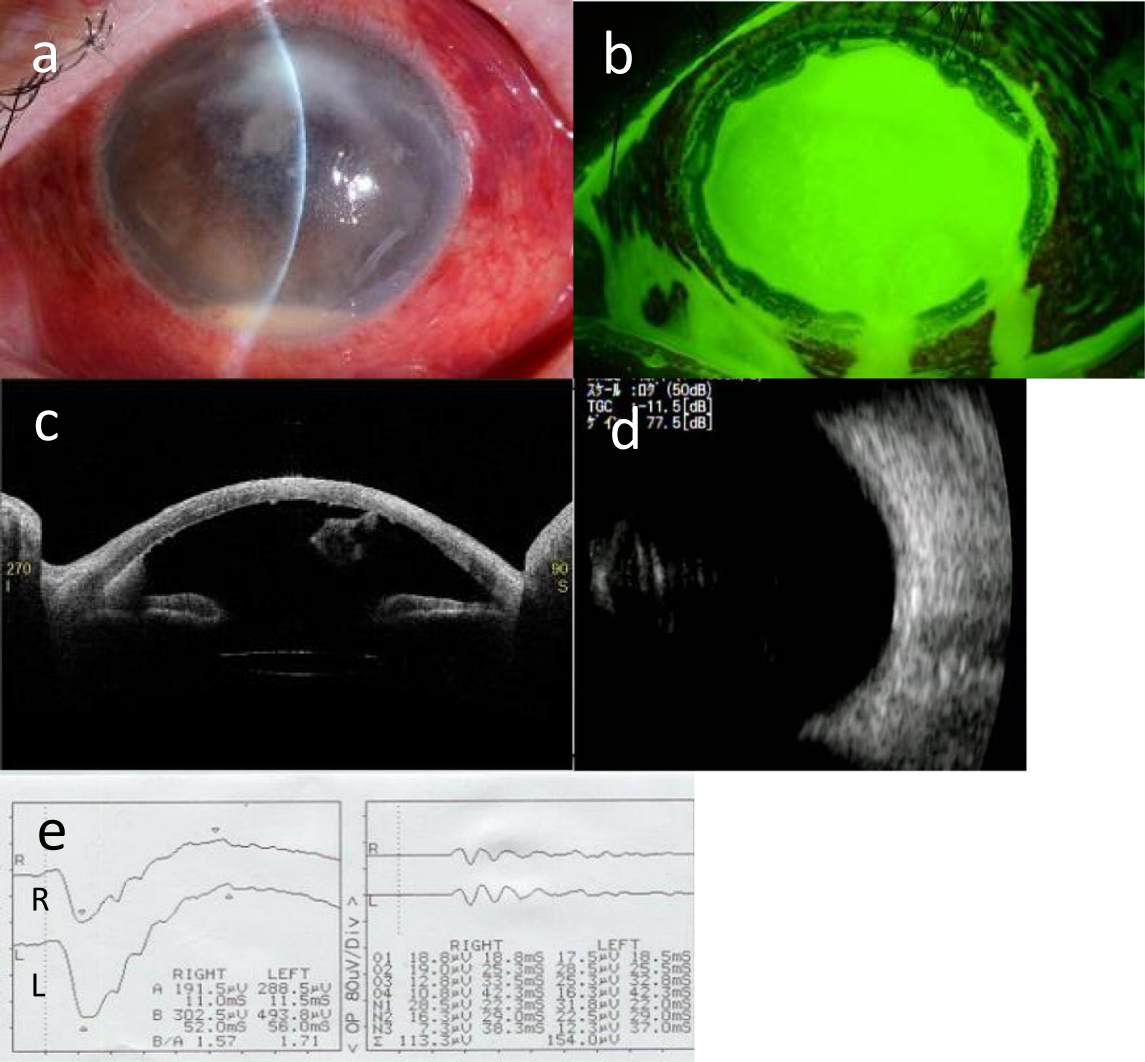
**a** Slit-lamp examination on the patient’s first visit. A white infiltrate on the upper cornea, hypopyon, anterior chamber fibrin formation, and ciliary hyperemia were observed. **b** A whole corneal epithelial defect was observed by fluorescein staining. **c** Anterior segment OCT with a CASIA2 showed a hypopyon, anterior chamber fibrin deposits, and keratic precipitates. **d** Echography showed no evident vitreous opacity. **e** The waveform of full-field ERG had no amplitude reduction or latency delay

On initial examination in our department, her visual acuity in the left eye allowed her to count fingers at a distance of 0.3 ft. Slit-lamp examination showed a white focus on the upper peripheral cornea, hypopyon, anterior chamber fibrin formation, marked ciliary hyperemia, and whole epithelial defects in the left eye (Fig. 1a,b). Anterior segment optical coherence tomography (OCT; CASIA2, Tomey) also revealed hypopyon, anterior chamber fibrin formation, and keratic precipitates (Fig. 1c). As echography showed no evident vitreous opacity, we speculated that infection had not advanced to the posterior chamber at this point (Fig. 1d). The full-field electroretinogram (ERG) waveform had no amplitude reduction or latency delay, which suggested that retinal function was not impaired by the infection (Fig. 1e). She was emergently hospitalized for infectious keratitis, and anti-bacterial treatment was continued.

The corneal scraping smear was positive for filamentous fungi and *Fusarium* species were detected by aqueous humor polymerase chain reaction (PCR). Antifungal susceptibility tests were performed and the MIC values were as follows: > 16 μg/ml for Micafungin, > 64 μg/ml for Flucytosine, > 64 μg/ml for Fluconazole, > 16 μg/ml for Miconazole, > 8 μg/ml for Itraconazole, 4 μg/ml for Voriconazole, and 2 μg/ml for amphotericin B. Thereafter, her treatment was shifted to anti-fungal therapy 2 days after hospitalization. The patient received topical 1% voriconazole hourly, 1% natamycin ointment 6 times daily, and intravenous liposomal amphotericin B injection (125 mg/day), whereas 0.1% betamethasone was replaced with 0.1% fluorometholone, and 0.1% tacrolimus was withdrawn. Adherence to topical medical therapy was checked by nursing staff by filling in the list of eyedrops.

**Fig. 2:**
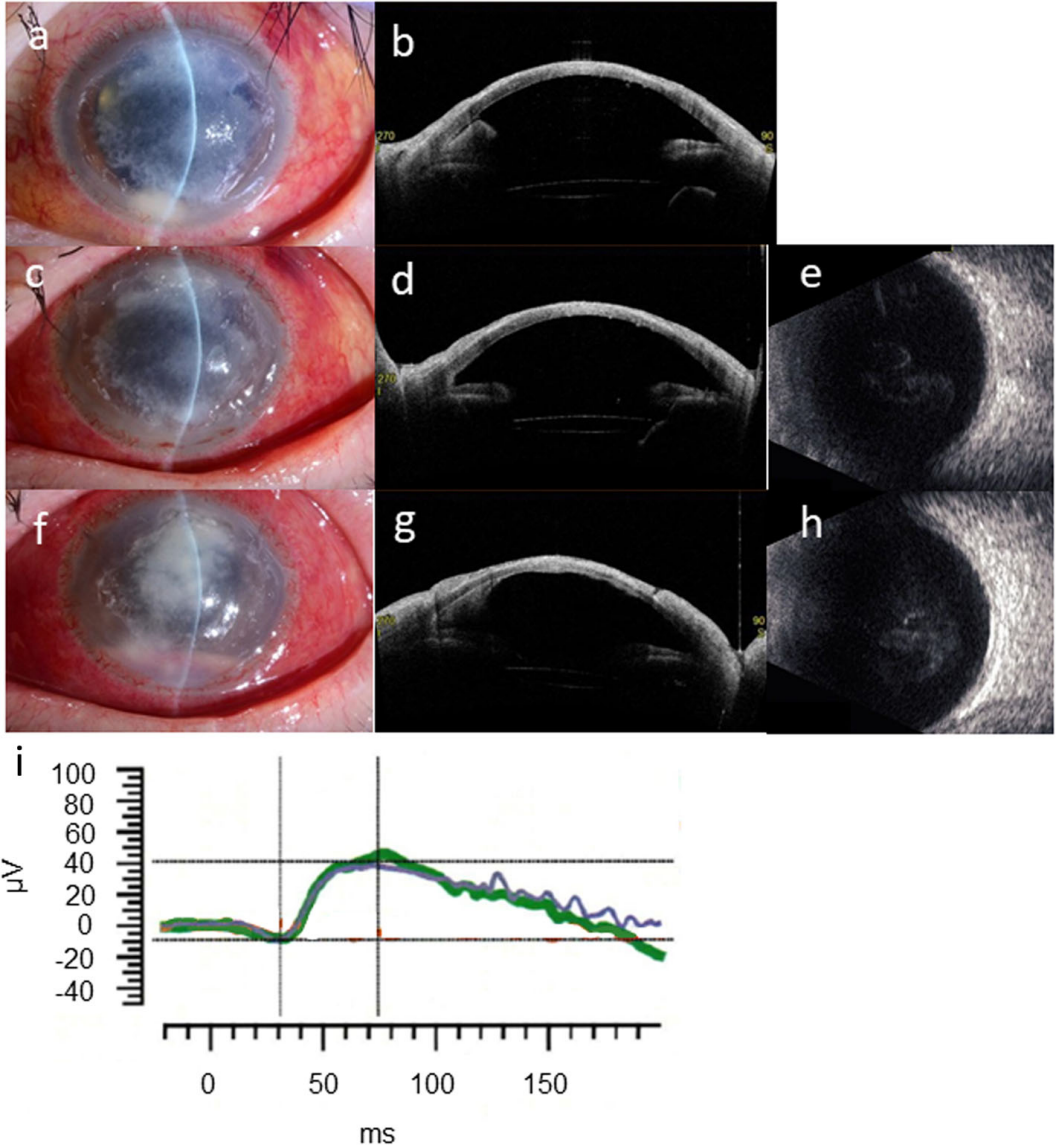
**a** The corneal infiltrate shrank and ciliary hyperemia improved 2 days after the onset of anti-fungal therapy. **b** Anterior segment OCT demonstrated decreased hypopyon and anterior chamber fibrin deposition. **c** and **d** The day after intravenous tocilizumab injection, aggravation of ciliary hyperemia was noted, although the hypopyon decreased due to anterior chamber irrigation. **e** Vitreous opacity emerged on echography 2 days after tocilizumab infusion. **f** and **g** Slit-lamp examination and anterior segment OCT 10 days after tocilizumab infusion. The range of corneal infiltration increased and the hypopyon worsened. **h** Hyperechogenic foci on B-scan ultrasonography also worsened. **i** Reduction of both a-wave and b-wave amplitude was confirmed on full-field ERG 9 days after tocilizumab administration

Two days after the onset of anti-fungal therapy, anterior inflammation improved, as characterized by a smaller corneal infiltrate and improved ciliary hyperemia; in addition, anterior segment OCT demonstrated a smaller hypopyon and anterior chamber fibrin deposits (Fig. 2a,b). However, after the patient’s monthly intravenous tocilizumab injection was administered 7 days after hospitalization (anterior chamber irrigation was performed on the same day), signs of recurrent inflammation were observed. Ciliary hyperemia markedly worsened, and vitreous opacity emerged on echography the day after tocilizumab injection (Fig. 2c–e).

As the initial response to anti-fungal therapy was good, and the vitreous opacity appeared to be a transient effect of tocilizumab application, therapy was continued without modification. However, 17 days after hospitalization, the degree of corneal infiltration, hypopyon, and vitreous opacity worsened, which suggested progression to endophthalmitis (Fig. 2f–h). In addition, both the a-wave and b-wave amplitude of the full-field ERG diminished, which suggested malfunction of the retina caused by advancement of the infection to the posterior segment (Fig. 2i). Given that the infection worsened in spite of conservative therapy, we determined that surgical treatment was required. After obtaining informed consent from the patient, penetrating keratoplasty (PKP) combined with vitrectomy was performed 19 days after hospitalization.

**Fig. 3:**
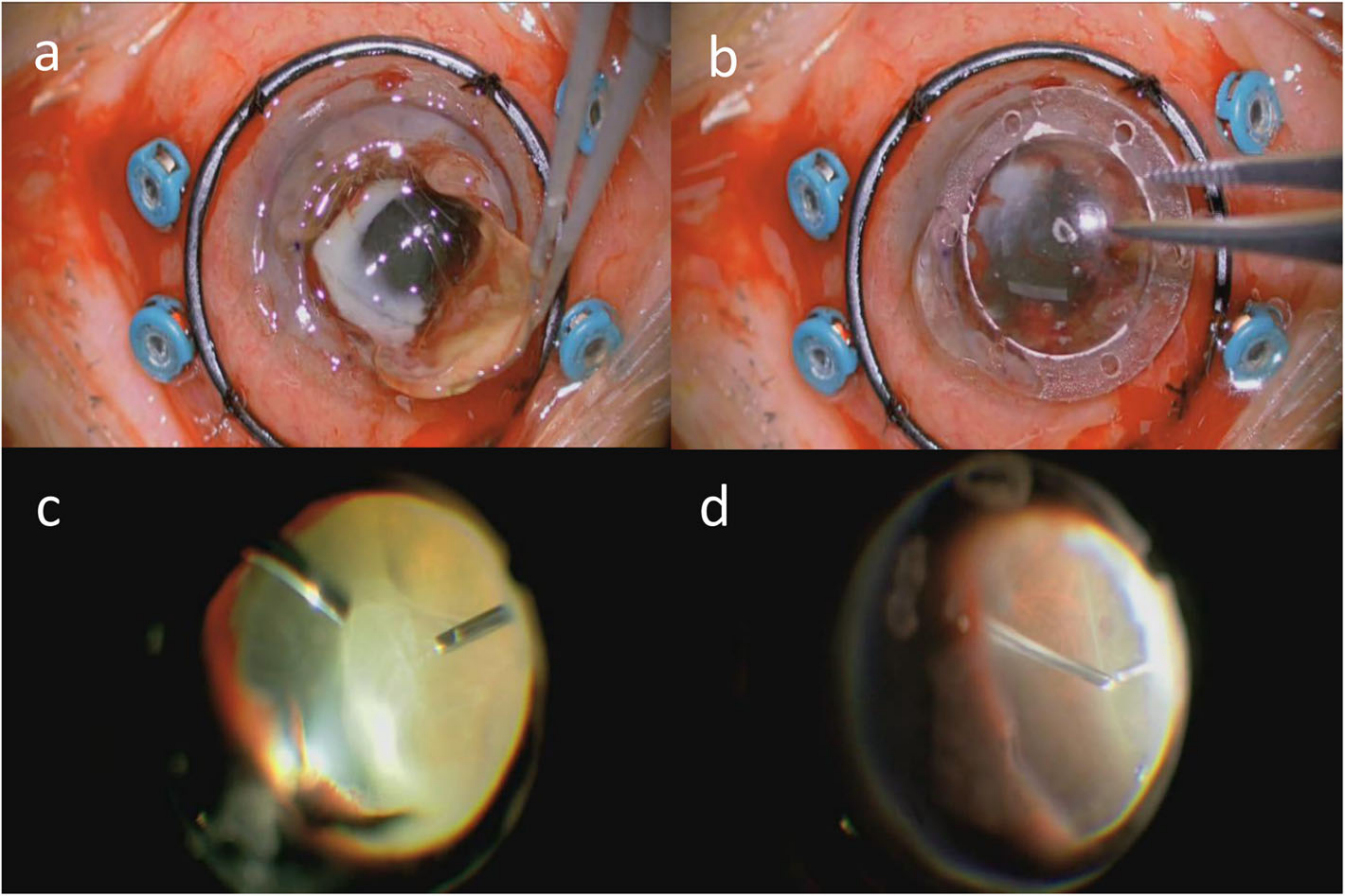
**a** A white focus at the rear of the IOL became evident after the IOL was removed together with the lens capsule. **b** A temporary corneal prosthesis was mounted. **c** Fundus examination revealed dense white vitreous opacity. **d** Dot hemorrhages and white foci (not pictured) were sparsely distributed on the peripheral retina

We used an intraocular infusion solution containing 10 mg/500 ml voriconazole. The host cornea was punched out with a 7-mm trephine. When the intraocular lens (IOL) was removed together with the lens capsule, a white focus ranging from the pupillary area to the back of the temporal iris became evident (Fig. 3a). After mounting the temporary corneal prosthesis (DORC, Fig. 3b), we performed a 25-gauge 4-port vitrectomy using the Constellation Vitrectomy System. The white focus in the pupillary area was removed during the anterior vitrectomy. Fundus examination revealed dense white vitreous opacity which was removed during the core vitrectomy and shaving (Fig. 3c). However, the focus in the vitreous had not adhered to the retina. A small white focus and dot hemorrhages were sparsely distributed on the peripheral retina (Fig. 3d). After removing the vitreous opacity completely, we removed the temporary corneal prosthesis and sutured in a frozen donor graft (7.75 mm in diameter). Pathological examination of the removed cornea by PAS and Grocott staining revealed filamentous fungi in the stroma.

**Fig. 4:**
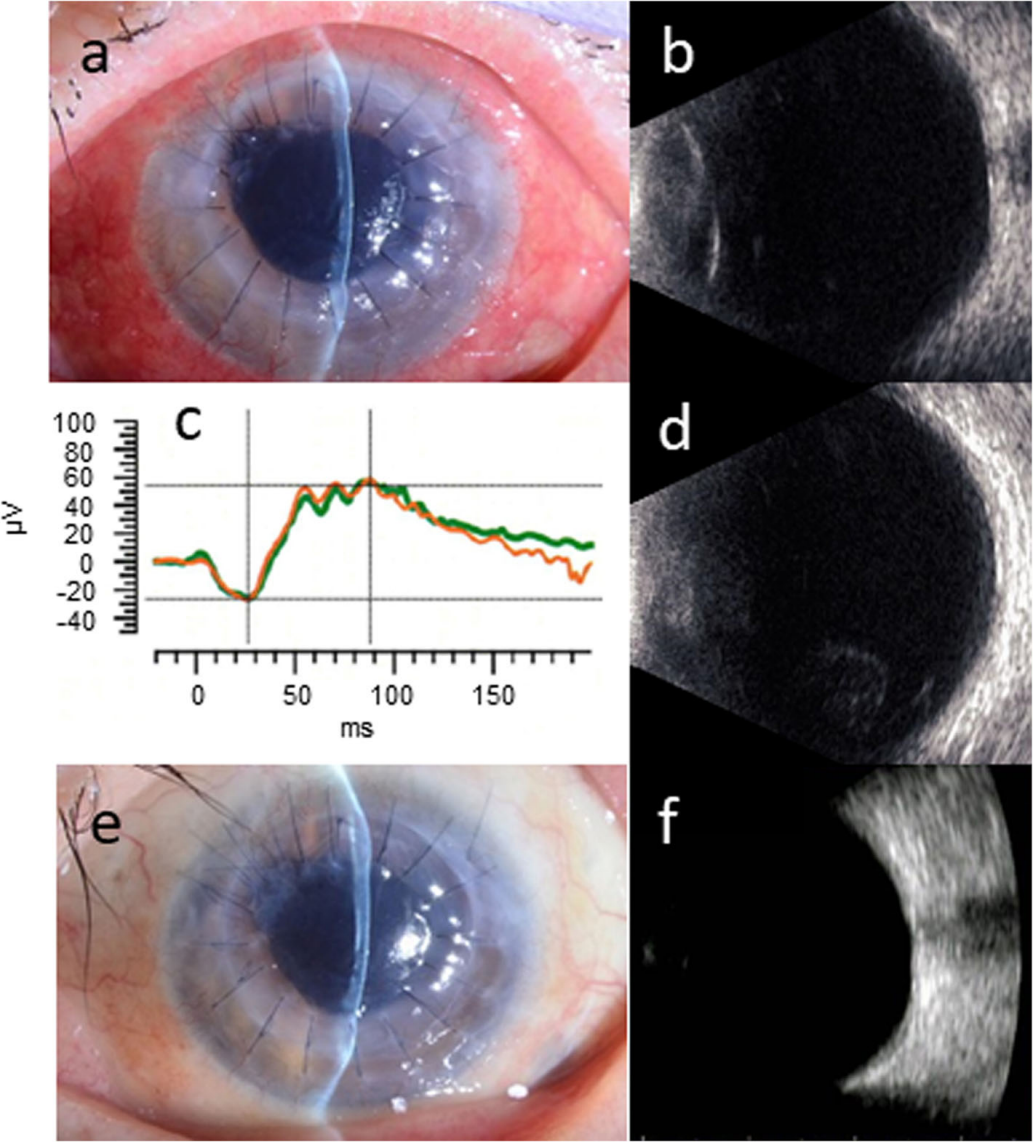
**a** Hypopyon and corneal infiltration were both absent 3 weeks after the surgery. (**b** and **c**) Vitreous opacity was largely absent on B-scan ultrasonography, and the full-field ERG waveform acquired at the same time had no amplitude reduction or latency delay. **d** Although mild vitreous opacity was confirmed on ultrasonography 4 days after tocilizumab infusion, it spontaneously improved. **e** and **f** There were no signs of infection relapse in the slit-lamp examination, and no vitreous opacity on B-scan ultrasonography 4 months after the surgery

The hypopyon and corneal infiltration were completely absent 3 weeks after the operation (Fig. 4a). The vitreous opacity was nearly absent by echography, and the waveform of the full-field ERG normalized at the same time (Fig. 4b,c). Although mild vitreous opacity emerged 4 days after the monthly intravenous tocilizumab injection (3 weeks after the operation), it showed spontaneous remission (Fig. 4d). As signs of inflammation improved, the amphotericin B infusion was discontinued and topical voriconazole and natamycin were both reduced to 4 times daily 1 month after the operation. The patient was discharged 2 months after the operation.

When she came to our department 4 months after the operation, the visual acuity of her left eye was 20/100. We observed neither relapse of infection nor vitreous opacity on echography (Fig. 4e,f). Optical PKP and IOL suture were performed 1 year after the initial therapeutic PKP combined with vitrectomy. The patient continues to visit our office (July 2020), and is doing well. The visual acuity of her left eye at her most recent visit was 30/100.

## Discussion and conclusions

We report a case of *Fusarium* infection complicating rheumatic keratitis in a patient receiving regular infusions of tocilizumab for rheumatoid arthritis; the infection took an atypical course and acutely progressed to endophthalmitis. To our knowledge, this is the first report of a case of *Fusarium* keratitis which acutely progressed to endophthalmitis during tocilizumab infusion.

The patient’s initial response to anti-fungal therapy was good; however, vitreous opacity rapidly emerged after the first tocilizumab infusion. Mild vitreous opacity also emerged after the second tocilizumab administration. Although we cannot draw any definitive conclusions, these observations suggest that tocilizumab may have been the aggravating factor for the fungal infection.

Although progression of fungal keratitis is generally indolent, the inflammation in the present case progressed acutely. There are several case reports of *Fusarium* keratitis progressing to endophthalmitis, but few studies dealing with large populations. One study included 159 *Fusarium* keratitis patients [4], of whom 10 patients had infections that progressed to endophthalmitis. In those cases, hypopyon and endothelial plaques emerged 10 to 40 days after corneal infiltration. Compared with the previously published cases, the present case took an extremely acute course, as hypopyon emerged only two days after corneal infiltration appeared. Several factors—including epithelial defect due to rheumatoid arthritis-associated corneal ulceration, steroid administration, and susceptibility to infection arising from tocilizumab infusion—may explain these observations.

One might speculate that steroid discontinuation could be the cause of ocular inflammation worsening. However, ocular inflammation mainly aggravated five days before the admission to our department, when oral prednisolone was started and the administration frequency of topical steroids was increased. Moreover, it did not acutely aggravate after three days before the visit to our department, when oral prednisolone was withdrawn and the administration frequency of topical steroids was decreased. Additionally, ocular inflammation improved two days after the onset of anti-fungal therapy, when the topical steroid was changed to a milder type. These observations suggest that steroid withdrawal is at least not the main factor for ocular inflammation aggravation.

The present case was characterized by acute progression to ophthalmitis during tocilizumab administration. Tocilizumab is an anti-IL-6 receptor antibody used for the treatment of autoimmune diseases, including rheumatoid arthritis. It mediates anti-inflammatory effects by inhibiting the binding of IL-6 to its receptor. As it also has an immunosuppressive effect, complications associated with its use include pneumonia (including *Pneumocystis* pneumonia) and tuberculosis.

Few studies have examined the relationship between fungal keratitis and IL-6. In a study which investigated the cytokine milieu in the tears of patients with fungal keratitis, higher levels of IL-6 were reported than in the tears of healthy individuals [6]. Application of inactivated *Fusarium solani* hyphae to cultured human corneal epithelial cells upregulated the secretion of IL-6 via activation of Toll-like receptors [7]. These studies indicate that IL-6 is upregulated in the anterior portion of the eye in response to fungal infection. However, IL-6 activity in this context is unknown. During infection with the bacterial pathogen *Streptococcus aureus*, application of IL-6 to the cornea of IL-6 knockout mice improved the symptoms of infection and reduced the number of bacteria to 1/3 the pre-treatment level [8]. Therefore, IL-6 may have anti-bacterial activity. IL-6 promotes elastase and free radical production by neutrophils, suggesting that it may inhibit bacterial infection via neutrophil activation. As neutrophils are the main cells that infiltrate the cornea in response to fungal infection, IL-6 inhibition by tocilizumab may aggravate inflammation through blockade of neutrophil activation.

To our knowledge, this is the first report of a case of fungal keratitis of which tocilizumab infusion was speculated to be an aggravating factor. There are some past studies describing tocilizumab as a risk factor for the onset and progression of systemic cryptococcosis and bacterial infection [9–11], so we speculate that progression of fungal keratitis is affected by tocilizumab infusion. However, the mechanisms of exacerbation and its incidence are not clear in the present case. Further study is needed to solve these problems.

In the present case, final visual acuity was relatively spared despite the acute course of the fungal infection. Past studies have indicated that *Fusarium* endophthalmitis developed from keratitis is associated with a poor prognosis, even after PKP. Therefore, early diagnosis and detection of endophthalmitis are needed, in combination with early surgical treatment to prevent the progression of endophthalmitis [4]. In the present case, culture of corneal scrapings and aqueous humor PCR were performed on the day of visit, which facilitated prompt diagnosis of *Fusarium* infection. PKP in combination with vitrectomy was performed at a relatively early stage, 10 days after vitreous opacity emerged on echography. We removed as much of the focus of infection as possible, using a temporary corneal prosthesis to improve visibility, before the infection progressed to the retina. In addition, intraocular drug migration was improved by vitreous removal. Our specific approach to treatment may be responsible for the recovery of the patient’s visual acuity.

One might say that a 10-day time-frame between Tocilizumab infusion and surgery is a considerable period of time for infection aggravation. We followed up this period by continuing conservative therapy, as the treatment seemed to be effective and fungal infection generally takes an indolent course. However, endophthalmitis worsened considerably at this relatively short period of time. The patient’s prognosis might have been better if the surgery had been performed as soon as possible, and this would be our future challenge.

Our experience suggests that tocilizumab infusion may make patients more vulnerable to ocular infection. Therefore, patients should be closely monitored for signs of ocular inflammation during tocilizumab administration, especially as rapid detection and thorough treatment may contribute to the preservation or recovery of visual acuity.

## Data Availability

All data generated and analyzed during this study are included in this article.

## Abbreviations

ERG: electroretinogram
IL-6: interleukin 6
IOL: intraocular lens
OCT: optical coherence tomography
PCR: polymerase chain reaction
PKP: penetrating keratoplasty
